# Medicines and vaccines supply chains challenges in Nigeria: a scoping review

**DOI:** 10.1186/s12889-021-12361-9

**Published:** 2022-01-05

**Authors:** Victory O. Olutuase, Chinwe J. Iwu-Jaja, Cynthia P. Akuoko, Emmanuel O. Adewuyi, Vishnu Khanal

**Affiliations:** 1 Department of Clinical Pharmacy and Pharmacy Practice, University of Jos, Jos, Nigeria; 2 Department of Nursing & Midwifery, Faculty of Medicine and Health Sciences, Stellenbosch University, Stellenbosch, South Africa; 3 Department of Nursing, Christian Service University College, Kumasi, Ghana; 4 Collaborative Genomics and Translation Group, Centre for Precision Health, School of Medical and Health Sciences, Edith Cowan University, Joondalup, Western Australia 6027 Australia; 5 Nepal Development Society, Butwal, Nepal

## Abstract

**Background:**

Medicines and vaccines supply chains represent critical systems for realising one of the major targets of the United Nations’ third Sustainable Development Goals (SDGs)—access to safe, effective, quality, and affordable essential medicines and vaccines, for all. However, evidence suggests the system is confronted with several challenges in many low-medium income countries, including Nigeria. This scoping review aims to summarize the available evidence on the challenges of medicines and vaccines supply chain system in Nigeria.

**Results:**

We searched relevant databases including Scopus and Web of Science for studies published between January 2005 and August 2020 on the challenges associated with medicines and vaccines supply chain systems in Nigeria. Our findings implicate several factors including difficulty with medicines or vaccines selection, procurement, distribution, and inventory management. Others included poor storage infrastructure, financial constraints, insecurity, transportation challenges, inadequate human resources, weak, or poorly implemented policies. These challenges mostly resulted in stock-outs of essential medicines which notably got worsened during the current COVID-19 pandemic.

**Conclusion:**

Our study is a wake-up call on the need to prioritise the critical sector of the supply chain systems for medicines and vaccines in Nigeria. Effective implementation of existing policies, improved security, strengthening of the health system through adequate budgetary allocations, and provision of infrastructure including regular availability of electricity are keys to surmounting the challenges and improving access to medicines or vaccines in Nigeria.

## Background

One of the major targets of the United Nations’ third Sustainable Development Goals (SDG), is to ensure access to safe, effective, quality, and affordable essential medicines and vaccines, for all [1]. This target is critical to achieving universal healthcare coverage just as effective health product supply chains are indispensable in ensuring access to quality medicines and vaccines [2]. Health product supply chains assure consistent availability of high-quality medicines, vaccines, and health products at health service delivery points in the most cost-effective and timely manner [3]. A functional health product supply chain system is indeed the backbone of quality healthcare services [4, 5]. The phenomenon not only guarantees the delivery of appropriate health products to the end-users, but it also ensures that health system planners receive critical information on the need, demand, and consumption of products, thus, contributing to better service delivery [3, 6].

The importance of supply chain management is widely acknowledged, however, access to quality essential medicines in developing countries including Nigeria continues to be a challenge [2, 6]. Challenges associated with medicine supply chain, in Nigeria, have been identified in the literature ranging from poor infrastructure, weak policy or regulatory implementation and quality compromised by substandard or counterfeit medicines [4]. Other challenges such as stock-outs, poor supply chain practices (e.g., poor inventory, poor forecasting, etc.), and inadequate human resources, amongst other factors, have been reported [3, 7–11]. To mitigate some of these challenges and improve the efficiency of medicines supply chains in Nigeria, some strategies have been implemented including the development of policies and programs such as the National Drug Policy, Nigeria Supply Chain Policy for Pharmaceuticals, National Drug Distribution Guidelines, regulation of human resources development, and engagement of professionals or personnel with relevant skills [12]. Despite these strategies, the supply chain system, in Nigeria, remains weak and inefficient [2].

So far, some studies, aimed at identifying the challenges associated with the supply chain systems for medicines or vaccines, have been conducted in Nigeria [2, 3, 7, 9–11]. However, to the best of our knowledge, no study has systematically reviewed the challenges either in the form of a scoping or a systematic review. It is important that these challenges are reviewed, pooling all the evidence, to facilitate the development of robust interventions. This study, thus, summarizes available evidence on the challenges of medicines and vaccines supply chain system in Nigeria. The study is aimed at a comprehensive review of challenges often ecountered in medicine and vaccine supply chain systems in the country. Consequently, we have chosen a scoping review, against a systematic review since the latter addresses precise questions, using a more predefined set of outcomes [13]. Also, while medicines and vaccines are both medicinal products that could be used for treatment or prevention of diseases, respectively, the supply chain of vaccines requires cold storage while medicines do not, except for some medications such as insulin. Hence, where appropriate, we have attempted to highlight supply chain challenges that are peculiar to vaccines. Findings in this study contribute to a better understanding of the subject, identify gaps in knowledge for future studies, as well as provide current evidence for policymakers on the challenges of medicines’ supply chain systems in Nigeria.

## Methods

This scoping review was conducted following the Joanna Briggs Institute methodology for scoping reviews [14]. We conducted a knowledge synthesis of existing research on the challenges associated with medicines and vaccines supply chains between 2005 and 2020 in Nigeria. This scoping review aims to answer the question of ‘what are the challenges of medicines and vaccines supply chain systems in Nigeria’?

### Search strategy

The search strategy for this study was decided and documented by VOO and CJI. The search strings were decided and extracted based on some preliminary articles from journals such as the Journal of Pharmaceutical Health Services Research and Vaccine [2, 15]. Searches were conducted in the Scopus and Web of Science (WOS) databases. These databases have large abstracts and citations covering numerous academic publications such as scientific journals, books, and conference proceedings. Moreover, the databases have a rich collection of research output in the field of medicine and health sciences coupled with a dynamic and flexible search engine for retrieving articles.

Since the main aim of this review was to extract empirical evidence on the challenges of medicine and vaccine supply chains in Nigeria, the search strategy was focused on identifying original research publications, reviews of original research, and case studies, and included all publications in these categories, both published or in the press, open or standard access options. We observed a rise in publication on medicine supply chain management from 2005 to 2020. Using the search terms, the databases were searched by titles, keywords, abstracts, and indexed keywords as shown in Table 1. The search was done in the English language and limited to studies carried out between January 2005 and August 2020. The search strings and keywords in Table 1 were applied first to medicines and subsequently replicated for vaccines. All searches were carried out in August and September 2020 and documented in an Excel workbook. An additional search was conducted in March 2021.

**Table 1:** Search strings and keywords

SN	Variable	Search Items
1	Medicine supply chain challenges	Medicine supply Nigeria
		Supply chain of medicines Nigeria
		Challenges of medicine supply Nigeria
2	Medicine selection challenges	Medicine selection Nigeria
		Challenges of medicine selection Nigeria
3	Medicine Quantification challenges	Medicine quantification Nigeria
		Challenges of medicine quantification Nigeria
4	Medicine Procurement challenges	Medicine procurement Nigeria
		Challenges of medicine procurement Nigeria
5	Medicine Distribution challenges	Medicines distribution Nigeria
		Challenges of medicine distribution Nigeria
6	Medicine Storage challenges	Medicine’s storage Nigeria
		Challenges of medicine storage Nigeria
7	Medicine Inventory management challenges	Inventory management of medicines Nigeria
		Challenges of inventory management of medicines Nigeria

### Duplicate Screening

All bibliographic citations extracted from Scopus were downloaded in a comma-delimited (CSV) format. VOO coordinated the search strategy, combined all CSV files into one file and used the remove duplicate function in Excel to remove 239 duplicates out of a total of 991 downloaded citations. We found a total of 1,844 relevant citations from the Web of Science database. Web of science has the feature of combining search results and removing duplicates. This feature was used to remove 514 duplicates. A total of 2,082 citations (including their titles, journal, year of publication, abstracts, keywords, and authors) was imported into Rayyan, a web-based systematic review software [16] for further duplicate screening and subsequent screening of titles and abstracts based on inclusion and eligibility criteria.

### Inclusion and eligibility criteria

A two-stage screening process was carried out as required by the scoping review methodology set in Joanna Briggs Institute Manual for Evidence Synthesis [17]. Articles that qualified for inclusion focused on at least one of the two main variables shown in Table 2. The studies had to be original research, review of original research or case published in English between 2005 and 2020. Conceptual papers describing one or more of the study variables were excluded. We applied the inclusion and eligibility criteria (Table 2), first in the selection of articles for medicines supply chain challenges and subsequently, vaccines supply chain challenges.

**Table 2:** Inclusion and eligibility criteria for this review

SN	Criteria	Explanation
1	Medicine supply chain challenges	The article should focus on medicine supply chain challenges
2	Medicine selection challenges	The article should focus on medicine selection challenges
3	Medicine Quantification challenges	The article should focus on medicine quantification challenges
4	Medicine procurement challenges	The article should focus on medicine procurement challenges
5	Medicine distribution challenges	The article should focus on medicine distribution challenges
6	Medicine storage challenges	The article should focus on medicine storage challenges
7	Medicine inventory challenges	The article should focus on medicine inventory challenges
4	Nigeria	The article should focus on medicine supply chain challenges, medicine selection, procurement, distribution, storage and inventory management challenges in Nigeria.
5	Study period	The article should cover 2005 and 2020
6	Study type	The article must be an original empirical study, review of original research or a case that focuses on medicine supply chain challenges

### Title, abstract and full-text screening

**Fig. 1:**
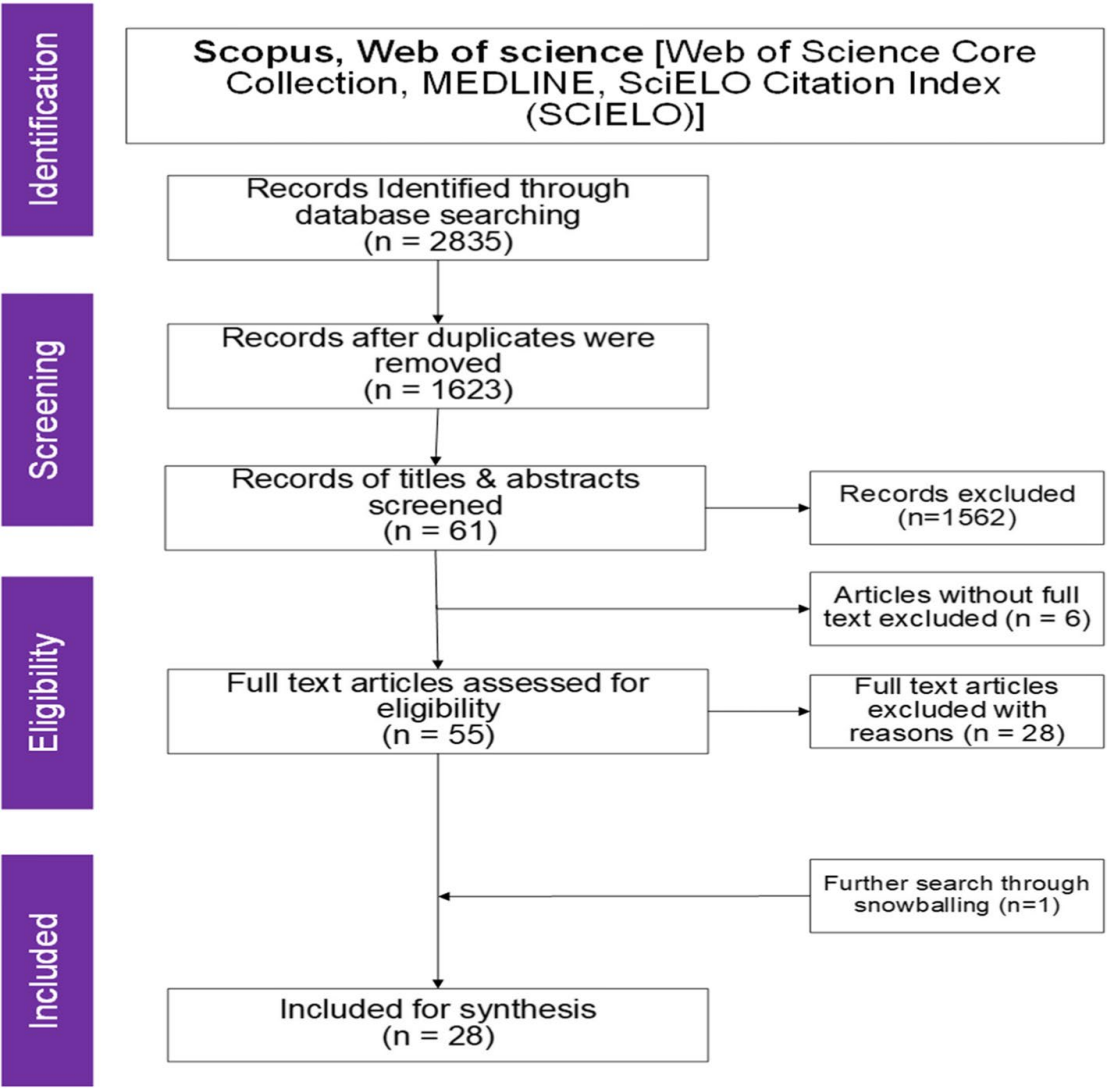
PRISMA flowchart of the study selection process

After screening and removing 459 duplicates via Rayyan, there were 1623 unique articles left for title and abstract screening. To avoid bias, the BLIND-review feature in Rayyan was activated for all authors to conduct an independent review of titles and abstracts based on the inclusion criteria. After this screening, there were initial 45 conflicts. Reviewers met to resolve these conflicts following the simple majority rule of two out of three reviewers. It, therefore, followed that two out of three reviewers' decision on inclusion or exclusion was affirmed. The full texts of 55 screened articles were thereafter attached to the citations already imported into Rayyan and were further reviewed for inclusion. Twenty-eight full texts were found irrelevant to the scoping review questions or objective and were thus excluded (see details in Table 3). Twenty-seven full-text articles were finally included for analysis (see details in Table 4). Through further Google scholar search, one relevant article was found and was included in the final list of 28 articles for analysis. Figure 1 presents a flow chart of the article selection process in this study.

**Table 3:** Excluded full texts based on eligibility criteria

SN	Authors	Title	Year	Reason
1	(Millar et al., 2014)	Patterns and predictors of malaria care-seeking, diagnostic testing, and artemisinin-based combination therapy for children under five with fever in Northern Nigeria: a cross-sectional study	2014	Irrelevant because it does not focus on medicine supply chain challenges
2	(Unger et al., 2014)	Treating diarrhoeal disease in children under five: the global picture	2014	Irrelevant because the full text does not capture study variables
3	(Mangham-jefferies et al., 2014)	What determines providers’ stated preference for the treatment of uncomplicated malaria?	2014	Irrelevant because the full text does not capture study variables
4	(Palafox, n.d.)	Mapping the private commercial sector distribution chain for antimalarials in six low-income countries in Africa and South East Asia	2014	Irrelevant because the full text does not capture study variables
5	(Obitte et al., 2009)	Survey of drug storage practice in homes, hospitals, and patent medicine stores in Nsukka, Nigeria	2020	Irrelevant because the full text does not capture study variables
6	Babalola, Stella and Lawan, Umar	Factors predicting BCG immunization status in northern Nigeria: a behavioral-ecological perspective	2009	Irrelevant because the full text does not capture study variables
7	Urban, Boris	Interventions to increase the distribution of vaccines in Sub-Saharan Africa: a scoping review	2019	Irrelevant because the full text does not capture study variables
8	(Karp et al., 2015)	Evaluating the value proposition for improving vaccine thermostability to increase vaccine impact in low and middle-income countries	2018	Irrelevant because the full text does not capture study variables
9	(Bassey et al., 2018)	The global switch from trivalent oral polio vaccine (tOPV) to bivalent oral polio vaccine (bOPV): facts, experiences and lessons learned from the south-south zone; Nigeria, April 2016	2018	Irrelevant because the full text does not capture study variables
10	(Odume, 2020)	Taking tuberculosis preventive therapy implementation to national scale: the Nigerian PEPFAR Program experience	2020	Irrelevant because the full text does not capture study variables
11	(Griswold et al., 2018)	Evaluation of Treatment Coverage and Enhanced Mass Drug Administration for Onchocerciasis and Lymphatic Filariasis in Five Local Government Areas Treating Twice Per Year in Edo State, Nigeria	2018	Irrelevant because the full text does not capture study variables
12	(Petersen et al., 2017)	Surveillance for falsified and substandard medicines in Africa and Asia by local organizations using the low-cost GPHF Minilab	2017	Irrelevant because the full text does not capture study variables
13	(Mangham-jefferies et al., 2015)	Mind the gap: knowledge and practice of providers treating uncomplicated malaria at public and mission health facilities, pharmacies and drug stores in Cameroon and Nigeria	2015	Irrelevant because the full text does not capture study variables
14	(Rao et al., 2017)	Immunization supply chains: Why they matter and how they are changing	2017	Irrelevant because the full text does not capture study variables
15	(Wright et al., 2017)	Improving iSC performance through outsourcing – Considerations for using third-party service providers to increase innovation, capacity, and efficiency	2017	Irrelevant because the full text does not capture study variables
16	(Ward & Kynvin, 2015)	Consumer-focused supply chains: a cross-case comparison of medicine appeal and acceptance in India, Uganda, and Nigeria	2015	Irrelevant because the full text does not capture study variables
17	(Molemodile et al., 2017)	Evaluation of a pilot intervention to redesign the decentralised vaccine supply chain system in Nigeria	2017	Irrelevant because the full text does not capture study variables
	(Osadebe et al., 2017)	Assessing Inactivated Polio Vaccine Introduction and Utilization in Kano State, Nigeria, April – November 2015	2017	Irrelevant because the full text does not capture study variables
18	(Hirsh Bar Gai et al., 2018)	Evaluating scenarios of locations and capacities for vaccine storage in Nigeria		Irrelevant because the full text does not capture study variables
19	(Bangura et al., 2020)	Barriers to Childhood Immunization in Sub-Saharan Africa: A Systematic Review	2020	Irrelevant because the full text does not capture study variables
20	(Daniel & Oladapo, 2006)	Default from tuberculosis treatment programme in Sagamu, Nigeria Default from Tuberculosis Treatment Programme in Sagamu, Nigeria	2006	Irrelevant because the full text does not capture study variables
21	(Ikoh et al., 2009)	The influence of “Stock Out” on health-seeking behaviour of low-income women in Uyo urban, Akwa Ibom State, Nigeria	2009	Irrelevant because the full text does not capture study variables
22	(Aina et al., 2017)	Preliminary results from direct-to-facility vaccine deliveries in Kano, Nigeria	2017	Irrelevant because the full text does not capture study variables
23	(Tougher et al., 2009)	The private commercial sector distribution chain for antimalarial drugs in Benin Findings from a rapid survey	2009	Irrelevant because the full text does not capture study variables
24	(Brien et al., 2018)	Treat the Pain Program Megan	2018	Irrelevant because the full text does not capture study variables
25	(Monath et al., 2016)	Yellow fever vaccine supply: A possible solution	2016	Irrelevant because the full text does not capture study variables
26	(Sutter & Cochi, 2019)	Inactivated Poliovirus Vaccine Supply Shortage: Is There Light at the End of the Tunnel?	2019	Editorial comment
27	(Oleribe et al., 2017)	Individual and socioeconomic factors associated with childhood immunization coverage in Nigeria	2017	Irrelevant because the full text does not capture study variables

**Table 4:** Included studies in the scoping review

SN	Title	Authors	Year	Journal	Journal Coverage	Type of paper	Focus	Methodology
1	Understanding Private Sector Antimalarial Distribution Chains: A Cross-Sectional Mixed Methods Study in Six Malaria-Endemic Countries	Palafox et al	2014	Public Library of Science (PLoS ONE)	Global	Original research	Medicines	Mixed methods
2	Differential determinants and reasons for the non- and partial vaccination of children among Nigerian caregivers	Sato 2019	2020	Elsevier	Global	Original research	Vaccines	Quantitative
3	Access to Routine Immunization: A Comparative Analysis of Supply-Side Disparities between Northern and Southern Nigeria	Eboreime Ejemai	2015	Public Library of Science (PLoS ONE)	Global	Original research	Vaccines	Quantitative
4	Procurement and Supply Management System for MDR-TB in Nigeria: Are the Early Warning Targets for Drug Stock Outs and Over Stock of Drugs Being Achieved?	Jatau et al	2015	Public Library of Science (PLoS ONE)	Global	Original research	Medicines	Quantitative
5	The availability, pricing and affordability of three essential asthma medicines in 52 low- and middle-income countries	Babar et al	2013	Springer International	Global	Original research	Medicines	Quantitative
6	Succeeding in New Vaccine Introduction: Lessons Learned From the Introduction of Inactivated Poliovirus Vaccine in Cameroon, Kenya, and Nigeria	Scotney et al	2017	The Journal of Infectious Diseases	Global	Original research	Vaccine	Quantitative
7	The status of hepatitis B control in the African region	Breakwell	2017	Pan African Medical Journal (PAMJ)	African	Original research	Vaccine	Quantitative
8	An evaluation of the cold chain technology in South-East, Nigeria using Immunogenicity study on the measles vaccines	Oli et al	2017	Pan African Medical Journal (PAMJ)	African	Original research	Vaccine	Quantitative
9	Reorganizing Nigeria's Vaccine Supply Chain Reduces Need For Additional Storage Facilities, But More Storage Is Required	Shittu et al	2016	Health Affairs	Global	Original research	Vaccine	Quantitative
10	Identifying barriers and sustainable solution to childhood immunization in Khana local government area of Rivers State, Nigeria.	Tobin-West C.I.; Alex-Hart B.A.	2011	International Quarterly of Community Health Education	Global	Original research	Medicine	Quantitative
11	Analysis of the Performance of Expanded Programme on Immunization (EPI) for Four Child Killer Diseases under the Military and Civilian Regimes in Nigeria, 1995-1999; 2000-2005	Obioha E.E.; Ajala A.S.; Matobo T.A;	2010	Studies on Ethno-Medicine	Global	Review	Medicine	Quantitative
12	Improving cold chain systems: Challenges and solutions	(Ashok et al., 2017)	2017	Vaccine	Global	Report	Vaccine	Qualitative
13	Vaccine wastage in Nigeria: An assessment of wastage rates and related vaccinator knowledge, attitudes and practices	(Wallace et al., 2017)	2017	Vaccine	Global	Original research	Medicine	Quantitative
14	Maternal reasons for non-immunisation and partial immunisation in northern Nigeria	Babalola S.	2011	Journal of Peadiatrics and Child Health	Global	Original research	Medicine	Quantitative
15	Assessment of community knowledge and participation in onchocerciasis programme, challenges in ivermectin drug delivery, distribution and non-compliance in Ogun State, southwest Nigeria	(Surakat et al., 2018)	2018	Infection, Disease & Health	Global	Original research	Medicine	Quantitative
16	How the quality of essential medicines is perceived and maintained through the pharmaceutical supply chain: A perspective from stakeholders in Nigeria	Amadi C., Tsui E.K.	2019	Research in Social and Administrative Pharmacy	Global	Original research	Medicine	Qualitative
17	The headache of medicines' supply in Nigeria: An exploratory study on the most critical challenges of pharmaceutical outbound value chains	(Aigbavboa & Mbohwa, 2020)	2020	Procedia Manufacturing	Global	Original research	Medicine	Quantitative
18	Poor performance of medicines logistics and supply chain systems in a developing country context: lessons from Nigeria	(Chukwu et al., 2018)	2018	Journal of Pharmaceutical Health Services Research	Global	Original research	Medicine	Mixed methods
19	Supply chain management of health commodities for reducing global disease burden	(Chukwu et al., 2017)	2017	Research in Social and Administrative Pharmacy	Global	Original research	Medicines	Quantitative
20	Medicine supply management in Nigeria: A case study of ministry of health, Kaduna state	(Mohammed & Magaji, 2007)	2008	Nigerian Journal of Pharmaceutical Sciences	African	Original research	Medicines	Mixed methods
21	Through service providers' eyes: Health systems factors affecting the implementation of tuberculosis control in Enugu State, South-Eastern Nigeria	Ogbuabor, D.C	2020	BMC Infectious diseases	Global	Original research	Medicines	Qualitative
22	Assessing Inactivated Polio Vaccine Introduction and Utilization in Kano State, Nigeria	(Osadebe et al., 2017)	2017	Journal of Infectious diseases	Global	Original	Vaccine	Qualitative
23	Transforming vaccines supply chains in Nigeria	(Sarley et al., 2017)	2017	Vaccine	Global	Report	Vaccine	Qualitative
24	Impact of vaccine stockouts on immunization coverage in Nigeria	(Gooding et al., 2019)	2019	Vaccine	Global	Original	Vaccine	Quantitative
25	Experiences from polio supplementary immunization activities in Anambra State, Nigeria	(Onyeka et al., 2014)	2014	Nigerian Journal of Clinical Practice	Nigerian	Original	Vaccine	Quantitative
26	Vaccine Storage and Handling Practices among routine immunization service providers in a metropolitan city of North-Central Nigeria	(H. A et al., 2013)	2019	Journal of Community Medicine and Primary Health Care	African	Original	Vaccine	Quantitative
27	Factors affecting vaccine handling and storage practices among immunization service providers in Ibadan, Oyo State, Nigeria	(Dairo & Osizimete, 2016)	2016	African Health Sciences	African	Original	Vaccine	Quantitative
28	The Challenges of Nigeria Vaccine Supply Chain, a Community of Practice Perspective	(Omole et al., 2019)	2019	International Journal of Research and Scientific Innovation (IJRSI)	Global	Original	Vaccine	Mixed methods

### Data extraction

Nine articles were allocated to three reviewers (VOO, CJI, and CKA.) each for data extraction. The reviewers extracted the data following the data extraction matrix as suggested in literature [13]. The extracted data included individual studies’ characteristics such as author(s), title, year of publication, journal, the focus of the paper, type of paper, methodology, and key findings based on objectives. These extracted data were captured in an Excel spreadsheet and later analyzed both quantitatively and qualitatively to answer the scoping review questions. It should, however, be noted that analyses did not extend to the quality of included articles as this is only a scoping review [13].

### Data summary and synthesis

Microsoft Excel was used to determine frequencies and simple percentages of the data. These were used to describe the nominal data extracted and to provide a summary of the data (see Table 5). Meanwhile, we performed a qualitative analysis of the included articles' key findings via Atlas.ti version 7. One of the authors (VOO) served as the administrator in this instance. The administrator created the project bundle and shared it with the other four authors. After an initial qualitative data analysis of key findings by the administrator, other authors further synthesized additional findings.

**Table 5:** General characteristics of included scoping reviews (n=28)

Characteristic	Number	Percentage (%)
*Total number of included articles*	**28**	**100%**
*Publication year*		
2005 - 2010	2	7%
2011 - 2015	7	25%
2016 - 2020	19	68%
*Journal coverage*		
Nigerian	1	4%
African	5	18%
Global	22	79%
*Focus of study*		
Medicine	14	50%
Vaccine	14	50%
*Type of paper*		
Original research	25	89%
Report	2	7%
Review	1	4%
*Methodology*		
Quantitative	19	68%
Qualitative	5	18%
Mixed method	4	14%

### Limitations of the methods

The possibility of missing additional evidence related to this subject is likely as articles published in unaccredited or un-indexed journals, considered predatory, may have been missed from the search strategy.

## Results

Database searches produced a total of 2,835 citations, out of which 27 articles were included in this review (see Figure 1 and Table 4). Analysis of the general characteristics of included articles (see Table 5) shows that about 68 per cent (n=19) of the articles were published between 2016 and 2020. The remaining articles were published between 2005 and 2015 (n = 9, 32%). Most of the reviewed papers were original research articles (n=25, 89%). Also, 79 per cent of the articles were published in international journals such as ‘Research in Social and Administrative Pharmacy’, and ‘Vaccine’. Fifty per cent of the articles focused on Medicine supply challenges (n =14 while the remaining 50% focused on vaccine supply challenges (n =14).

The frequency for the number of articles reporting each challenge was calculated (see Table 6). Issues relating to medicines or vaccines stockouts topped the list of challenges. Eighteen per cent of the articles (n=8) highlighted these challenges on stockouts, while 14 per cent of the articles (n=6) reported on human resource challenges, storage challenges and technical issues respectively. Eleven per cent of the articles (n=5) further reported on financial challenges, transportation and distributions challenges, policies, and SOPs challenges respectively. Issues on poor data management of medicines and vaccines supply were the least reported (7%, n=3 articles).

**Table 6:** Frequency of articles reporting each challenge

Theme	No of articles	Frequency of articles reporting each challenge
Human resource challenges	6	14%
Financial challenges	5	11%
Transportation and distributions challenges	5	11%
Policies and SOPs challenges	5	11%
Storage challenges	6	14%
Issues relating to medicines or vaccines stockouts	8	18%
Technical issues	6	14%
Poor data management of medicines and vaccines supply	3	7%

Key findings, answering the scoping review question, are summarized in Table 7, and presented in the sub-sections below.

**Table 7:** Summary of key findings from data synthesis

Theme	Key findings	Studies
Human resource challenges	Challenges experienced by pharmacists with the various aspects of the supply chain Lack of support for personnel involved in medicine logistics, inadequate personnel, lack of human resources as well as corruption, killing of personnel due to insurgency	(Chukwu et al., 2018) (Eboreime et al., 2015) (Chukwu et al., 2017) (Mohammed & Magaji, 2007) (Aigbavboa & Mbohwa, 2020)
Financial challenges	Lack of financial resources, Poor funding for vaccine supply	(Mohammed & Magaji, 2007) (Chukwu et al., 2018) (Sarley et al., 2017) (Omole et al., 2019) (Obioha et al., 2010)
Delay, transportation and distributions challenges	Delays in importation and difficulty in maintaining delivery vehicles. Distribution challenge due to delay in submission of inventory reports and submission of inaccurate inventory reports, insecurity during transportation of vaccines and logistics distance between manufacturer and Nigeria. Inability to monitor and maintain optimum temperatures for vaccines during transportation	(Palafox et al., 2014) (Jatau et al., 2015) (Omole et al., 2019) (Oli et al., 2017) (Ogbuabor, 2020)
Policies and Standard Operating Procedure (SOP) challenges	Inadequate implementation of medicine distribution policies, sub-optimal implementation of policies, non-adherence to policies	(Chukwu et al., 2017) (Amadi & Tsui, 2019) (Chukwu et al., 2018) (Scotney et al., 2017)
Infrastructure and storage challenges	Disruption of the supply chain through the destruction of storage facilities, inadequate storage facilities for ivermectin, inadequate cold storage facilities, inadequate ice-packs	(Aigbavboa & Mbohwa, 2020) (Surakat et al., 2018) (Shittu et al., 2016) (Ashok et al., 2017) (Sarley et al., 2017) (Ameen et al., 2013)
Issues including medicines or vaccines stockouts	Stock-outs, substandard medicines, shortage of vaccine stock and vaccine stock-outs, Regular stock-outs of essential medicines due to inefficient inventory management systems, equipment and corruption, Inadequate supply of vaccines	(Aigbavboa & Mbohwa, 2020) (Babalola 2011) (Sato, 2019) (Gooding et al., 2019) (Chukwu et al., 2018) (Obioha et al., 2010) (Mohammed & Magaji, 2007) (Breakwell et al., 2017)
Technical issues	Interruption of drug supplies, Unreliable vaccine supply, Inefficient procurement systems, Damaged products and packages, loss of potency of cold chain medical supplies, Irregular power supply and use of archaic technology in vaccine handling, inadequate ice blocks to maintain a cold chain	(Breakwell et al., 2017) (Ogbuabor, 2020) (Babar et al., 2013) (Dairo & Osizimete, 2016) (Ashok et al., 2017) (Onyeka et al., 2014)
Poor data management of medicines and vaccines supply	Poor procurement, incomplete forecasting, poor data collection, use and management, Poor reliability and availability of data for forecasting and decision making, Sub-optimal data on vaccine stock, Poor reliability and availability of data for forecasting and decision making	(Chukwu et al., 2018) (Omole et al., 2019) (Wallace et al., 2017)

### Question: What are the challenges of medicines and vaccines supply chain in Nigeria?

While medicine and vaccine supply chains are recognised, globally, as a key driver of access to medicines, evidence indicates that the system is often faced with many challenges. The objective of this scoping review was to summarize what is known about these challenges in Nigeria. In this review half of the studies (50%) reported on the challenges confronting medicine supply chains, while the other half (50%) reported the vaccine supply challenges in the country. The challenges were further categorized into themes as presented in Table 7.

### Human resource challenges

Professionally, pharmacists play substantial roles in several aspects of medicine supply chains, yet not without challenges in aspects such as forecasting, procurement, inventory control, distribution and logistics management information system as reported by some authors [8]. Lack of support for personnel involved in medicine logistics and killing of personnel due to insurgency, inadequate personnel, lack of human resources, as well as corruption, were some of the medicine supply chain challenges identified in some states in Nigeria [2, 3, 8, 18]. In a study to evaluate access to immunization services in the Southern and Northern regions of Nigeria, inadequate human resources and vaccines were reported at service delivery points [19].

### Financial challenges

An analysis of the expanded program on immunisation (EPI), indicated that there was poor coverage of the program due to shortage in vaccine supply and low funding for the program [20, 21]. Furthermore, corruption concerning funds meant for medicine supply, poor or inadequate funding, and lack of funds are some of the medicine and vaccine supply chain financial challenges, that have been reported by authors [2, 18, 20, 22, 23].

### Delay, transportation, and distributions challenges

Challenges related to delays in supply and operational logistics are commonly highlighted in studies [23–26]. For example, delay in importation and difficulty in maintaining the delivery vehicles in the supply of antimalarials were identified in a study in Nigeria [25]. Similarly, delay in the distribution of medicines for multi-drug resistant tuberculosis, due to delay in the submission of inventory reports, coupled with inaccurate reports and transportation challenges such as breakdown of vehicles were reported by some authors [24]. Inability to maintain optimum vaccine temperatures in delivery vehicles was also reported as a vaccine supply chain challenge in an evaluation of cold chain technology on measles vaccines [26]. A study reported the interruption of Tuberculosis (TB) drugs supply handled by logistics company, due to ineffective distribution of the medicines to the health facilities [27]. Insecurity during transportation of vaccines, inability to maintain the integrity of vaccines in the supply chain, and logistics distance between manufacturer and Nigeria were all identified as vaccine supply challenges in a recent study [23]. There are equally other challenges including damaged products and packages, unorganised supplies due to multiple distribution channels in the country which is associated with a high level of pilferage and loss on transit [2]

### Policy and standard operating procedure challenges

Issues around poor policy or policy implementation rank among major challenges confronting the medicine supply chain system in Nigeria. A recent qualitative study, for example, highlighted the challenges posed by the lack of stringent policies or weak implementation of existing policies on the distribution and quality of medicines in the country [4]. Additional evidence implicates weak supply chain practices and a poor system of regulation [2, 3]. Challenges such as poor procurement, incomplete forecasting, data collection and management, sub-optimal implementation of policies were further reported [2, 8]. It has been discovered that non-adherence to certain policies on vaccine administration led to excessive consumption exceeding supply which translated to Inactivated Poliovirus Vaccine (IPV) stockout in Nigeria between March 2015 and June 2016 [28].

### Infrastructure and storage challenges

Challenges bordering on infrastructure are equally highlighted in studies, for instance, in a survey among health workers, 62% of respondents mentioned inadequate storage facilities for medicines as one of the challenges encountered in the distribution of ivermectin [29]. Disruption of the supply chain through the destruction of storage facilities is similarly noted [3]. Substandard, inadequate vaccines storage facilities have also been identified especially in the local government areas of Nigeria [30]. Some of the challenges that affected cold chain performance were inadequate dry and cold chain storage facilities [22, 31]. A study on the storage practices of vaccines in a state in Nigeria reported that only 28.6% of the health facilities had functional thermometers in their stores [32].

### Issues including medicines or vaccines stockouts

Other challenges commonly encountered include stock-outs, substandard medicines, inadequate supply of vaccines, regular stock-outs of essential medicines due to inefficient inventory management systems [2, 3, 21, 33]. In a study on the impact of vaccine stockouts on the immunisation status of children, a lot of vaccine stockouts were reported [15]. Shortages and unreliable vaccine supply were reported as parts of the reasons for incomplete immunization and a barrier to immunisation uptake by children in some studies [20, 33, 34]. Incomplete immunisation schedules due to an inadequate supply of vaccines have also been reported [18].

### Technical issues

Suboptimal medicine infrastructure, substandard or use of archaic equipment in handling vaccines as well as poor monitoring of the required standard temperatures for the vaccines are among technical issues often encountered in the supply chains for medicines in Nigeria [3, 31]. Inadequate ice packs to maintain optimum temperatures in the cold chain was reported as a challenge during a study on Polio immunization challenges [35]. Lack of stable power supply which leads to variability in temperatures of the vaccines has been reported as one of the challenges of vaccine supply management in Nigeria [36]. Another study in 52-low-middle income countries which includes Nigeria to determine the availability of asthma medications discovered inefficient procurement systems for the asthma medications in these countries [37] Irregular supply of vaccines has also been mentioned as one of the challenges encountered in Hepatitis B vaccination [34]. Poor equipment and corruption are part of the identified challenges associated with medicine supply chains in Nigeria [2, 3, 21, 33].

### Poor data management of medicines and vaccines supply

Poor data collection, quality and use of data were some of the vaccine supply chain challenges reported in a study in Lagos state Nigeria [22]. Sub-optimal data on vaccine stock was also reported in a study on vaccine wastage in Nigeria [38]. Poor reliability and availability of data for forecasting and decision making were further reported [23].

### Rural-urban or regional differences

Table 8 summarises settings (rural, urban, or mixed residence) where the reviewed studies were conducted in Nigeria. Notably, studies focused specifically on rural-urban differences in medicine and vaccine supply chain systems or those comparing one region with another (north vs south, for example), are limited. Many of the reviewed studies used data from multiple centres across the country (or population-based surveys) [3, 8, 15, 20, 22–24, 28, 31, 33, 34, 37, 38], and thus, maybe nationally representative. We note that study settings (rural or urban) were rarely clearly described in many of the reviewed papers. Nonetheless, most of the data, including those from multiple centres, for example, the seven multi-drug resistant tuberculosis (MDR-TB) centres [24] were more likely to be from urban areas, and, thus, less likely to be nationally representative. This observation would mean some of the challenges reported in our study were probably underestimated as rural areas would normally be expected to experience even greater challenges associated with medicine and vaccine supply chain systems than urban centres in Nigeria. Where data from two or more local government areas (LGAs) were reported in the reviewed studies (Table 8), it may be expected that these cut across rural and urban areas in the respective states, since LGAs in Nigeria may have a mix of rural and urban centres. However, this is not necessarily the case in the present studies as some of the LGAs were indeed in urban settings, for example, the two LGAs studied in Kwara State (Ilorin East and Ilorin West) [32]. Overall, these findings suggest the need for future studies to prioritise rural-urban or regional differences in medicine and vaccine supply chain systems in Nigeria.

**Table 8:** Study Setting of included articles

Authors	Study setting
Palafox et al 2014	Urban
Sato 2019	Setting is nationally representative
Eboreime Ejemai, 2015	Four states, two Northern and two Southern states
Jatau et al,2015	Setting is nationally representative (All the seven MDR-TB Centres in Nigeria)
Babar et al, 2013	Data is nationally representative
Scotney et al, 2017	Data is nationally representative
Breakwell, 2017	Data is nationally representative
Oli et al, 2017	South-East, Nigeria
Tobin-West C.I.; Alex-Hart B.A., 2011	Khana Local Government, Rivers State
Obioha et al., 2010	Data is nationally representative
Ashok et al., 2017	Data is nationally representative
Wallace et al., 2017	Data is nationally representative
Babalola 2011	Northern Nigeria
Surakat et al., 2018	Eight local government areas in Ogun State
Amadi & Tsui, 2019	Four states, Enugu, Imo, lagos and Port-Harcourt
Aigbavboa & Mbohwa, 2020	Data is nationally representative
Chukwu et al., 2018	Data is nationally representative
Chukwu et al., 2017	Abuja, Nigeria
Mohammed & Magaji, 2007	Kaduna State
Ogbuabor, 2020	EnuguState
Osadebe et al., 2017	Kano State
Sarley et al., 2017	Data is nationally representative
Gooding et al., 2019	Data is nationally representative
Onyeka et al., 2014	Anambra State
Ameen et al., 2013	Two local Government areas in Kwara State, Nigeria
Dairo & Osizimete, 2016	Eleven Local Government areas in Ibadan
Omole et al., 2019	The data is nationally representative

## Discussion

This scoping review summarises the current body of knowledge on challenges associated with medicines and vaccines supply chain system in Nigeria. Our key findings implicate several challenges, particularly, those related to the key areas of medicines supply chain management in the country. Difficulty with medicine or vaccine selection, procurement, distribution, inventory management and storage infrastructure formed critical components of some of the challenges that mostly resulted in stock-outs of essential medicines in Nigeria. Challenges relating to medicines or vaccines stockouts were reported by the highest number of articles (n=8), while challenges relating to poor data management of medicines and vaccines supply were only reported by three articles. Furthermore, financial constraints, poor information management and inadequate human resources were identified as parts of the challenges confronting the supply chains for medicines in the country.

Generally, our study found that frequent breakdown of vehicles coupled with poor road network system contributed to delay in the distribution of medicines in Nigeria and the finding is consistent with reports from other low-middle income countries such as Malawi [39]. This is, however, not the case in high-income countries such as the United States of America, where distribution challenges identified were more related to the lack of coordinated distribution of drug supplies especially during this present COVID-19 pandemic [40].

Challenges related to inefficient inventory control systems were consistently identified in several of the reviewed studies contributing largely to regular stock-outs of medicines. This finding agrees with the results of a study on procurement challenges conducted in South Africa which similarly identified inefficient inventory control systems as parts of the factors contributing to shortages of medicines in the country [41]. Inadequate storage facilities, as well as an irregular power supply (necessary for maintaining cold chains vaccines), were common challenges reported in several articles reviewed in this study. These challenges could negatively impact the quality and efficacy of medicines and vaccines made available to patients. Our findings are consistent with those of an Ethiopian study which similarly reported inadequate storage facility and inability to maintain optimum temperature for the cold chain as parts of the challenges associated with medicines and vaccines supply chain system in the country [42]. Another study in Ethiopia also reported inadequate storage space for antiretroviral medicines and other HIV/AIDS-related products [43].

Weak policies, non-adherence and poor implementation of policies on medicines and vaccine supply identified in this review have also been identified in a systematic review on medicines management in India [44]. Insurgency has also been a major challenge identified in the medicines and vaccine supply chain in Nigeria and this was equally found in other countries around the world where insurgency have greatly impacted their healthcare delivery [45]. Other countries that reported a disruption in medicine and vaccine supplies were due to natural disasters and pandemics such as COVID-19 [40]. It is important to note that the challenges in Nigeria and other countries like Taiwan have been further complicated by the COVID-19 pandemic [46, 47]. Financial challenges, corruption and lack of human resources which are other challenges identified in this review have also been reported in Uganda [48].

## Conclusion/ Recommendations

This study provides a summary of the challenges associated with supply chain systems for medicines and vaccines in Nigeria. Our findings revealed several challenges which contributed to frequent stock-outs of essential medicines in the country. Stockouts would impact access to quality essential medicines thereby undermining efforts aimed at meeting one of the major targets of SDGs in Nigeria—access to safe, effective, quality, and affordable essential medicines and vaccines, for all. It is worth noting that the emergence of the COVID-19 global pandemic may have further complicated some of the challenges associated with medicines and vaccines supply chain in Nigeria. This suggests the need for creative context-specific approaches to addressing the challenges identified in our study. Moreover, our study highlights the need for more studies, especially, with regards to the rural-urban, or regional differences and in the context of the emergence of COVID-19 pandemic. Overall, the present study serves as a wake-up call to policymakers and regulators on the need to prioritise the critical sector of the supply chain system for medicines and vaccines in Nigeria. There is a need for effective strengthening of the system through adequate budgetary provision. Infrastructural development and regular availability of electricity supply are keys to the success of the supply chain system for medicines and vaccines in the country. Also, there is an urgent need for a deliberate effort aimed at effective implementation of relevant existing policies in the sector. This recommendation assumes greater importance given that a lack of stringent policy and weak implementation of existing policies were identified as major challenges in many of the reviewed studies.

## Data Availability

The datasets used and/or analysed during the current study are available from the corresponding author on reasonable request.
